# Implementation of microsurgery simulation in an ophthalmology clerkship in Germany: a prospective, exploratory study

**DOI:** 10.1186/s12909-022-03634-x

**Published:** 2022-08-03

**Authors:** Svenja Deuchler, Julia Scholtz, Hanns Ackermann, Berthold Seitz, Frank Koch

**Affiliations:** 1 Augenzentrum Frankfurt, Georg-Baumgarten-Straße 3, 60549 Frankfurt am Main, Germany; 2 Department of Ophthalmology, Saarland University Medical Center, 66421 Homburg/Saar, Germany; 3 Department of Ophthalmology, University Hospital, Goethe University Frankfurt, Frankfurt am Main, Germany; 4 Institute of Biostatistics, Goethe University Frankfurt, Frankfurt am Main, Germany

**Keywords:** Eyesi, Medical students, Microsurgery, Surgical simulator, Surgical skills, Undergraduate medical education, Virtual reality, Vitreoretinal surgery

## Abstract

**Background:**

Microsurgery is a growing field which requires significant precision and skill. Eyesi Surgical, which is usually introduced during residency or fellowship, is an ophthalmologic microsurgery simulator which allows users to practice abstract microsurgical skills and more specialized skills. The purpose of this study was to assess the inclusion of microsurgical simulation training during medical school.

**Methods:**

Seventy-nine German medical students in their 10^th^ semester of education completed up to two days of training on the simulator during their ophthalmology clerkship. They received an objective numeric score based on simulator performance and completed pre and post training subjective questionnaires.

**Results:**

There was no relationship found between students’ Eyesi Surgical performance scores and their specialty interests (*p* = .8). The majority of students (73.4%) rated their microsurgical skills to be higher after simulator training than before training (*p* < 0.001). 92.4% of students found the Eyesi Surgical to be a useful component of the ophthalmology clerkship. Objective scores from Navigation Training Level 1 showed that students achieved better results in the criteria categories of Completing Objects and Tissue Treatment than in the categories of Instrument and Microscope Handling. The mean Total Score was 25.7 (± 17.5) out of a possible 100 points.

**Conclusion:**

The inclusion of surgical simulation in the ophthalmology clerkship led to increased confidence in the microsurgical skills of medical students. Offering surgical simulation training prior to residency can help to expose students to surgical fields, identify those that have particular talent and aptitude for surgery, and assist them in deciding which specialty to pursue.

**Supplementary Information:**

The online version contains supplementary material available at 10.1186/s12909-022-03634-x.

## Background

Microscopic surgery is becoming more prevalent across a wide variety of medical specialties. The finessed technique required to successfully perform microscopic and often minimally invasive surgeries is a learned skill, but some individuals are better suited to the task than others. During their medical education, it is important for students be exposed to a variety of medical specialties so that they are able to choose a residency that suits both their interests and abilities. Practical experiences such as clinical rotations and simulated patient interactions can help to guide students in their decision making.

**Fig. 1:**
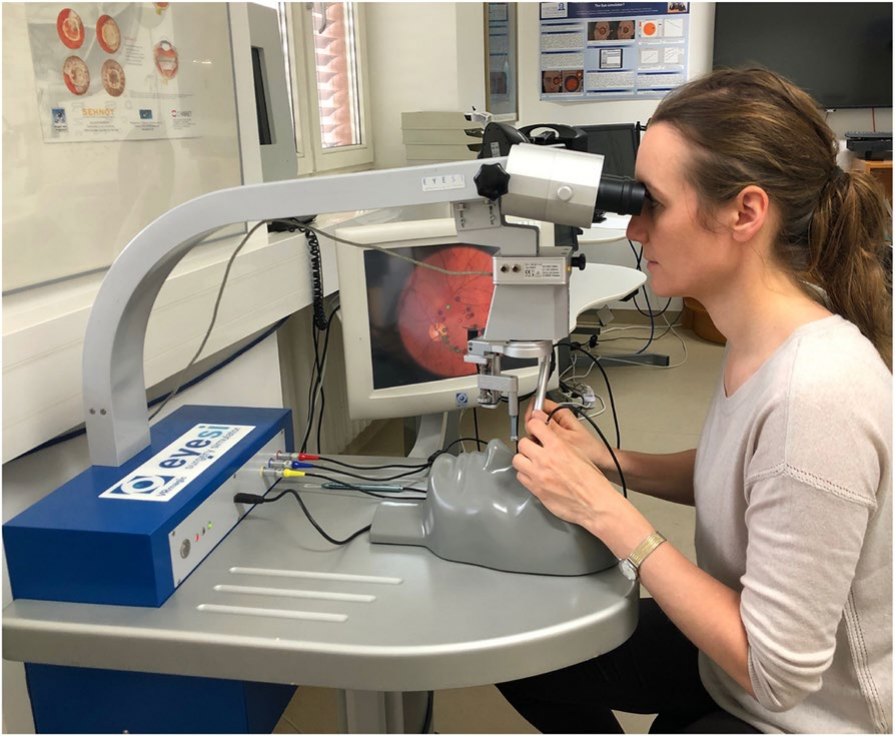
Eyesi Surgical simulator in use

The Eyesi Surgical simulator from Haag-Streit Simulation is a virtual reality simulation tool that enables trainees to practice microscopic intraocular surgery in a realistic simulated environment. The curriculum included with the device begins with basic skills practice and progressively advances users through anterior or posterior segment surgical tasks as skills and comfort increase. Eyesi Surgical allows individuals to improve their surgical skills and confidence without the risk of injuring a real patient. Eyesi utilizes a binocular operating microscope, surgical instruments, a patient with mechanical eye, and foot pedals for operating equipment (microscope, phaco, vitrectomy machine, and laser) to create the simulated surgical environment (Fig. 1). Trainees begin with abstract Basic Navigation training and progress through different levels on the simulator, ending with more complex clinical skill modules.

Our team has assisted in the development of simulators in ophthalmology, including diagnostic simulators for ophthalmoscopic and slit lamp examinations (Eyesi Direct, Indirect, Slit Lamp) and surgical simulators for cataract and vitreoretinal surgery (Eyesi Surgical). In recent publications, we assessed the introduction of the Eyesi Direct and Indirect ophthalmoscopy virtual reality simulators in the medical school curriculum [1, 2]. Virtual reality training of direct and indirect ophthalmoscopy is important from a teaching and learning standpoint. Even if an institution has enough real ophthalmoscope devices for all pupils, the option to examine each other assumes that all students are willing to have at least one pupil dilated. These peer examinations are generally performed on healthy eyes, which does not allow students to examine typical pathologies routinely.

The Eyesi Surgical simulator is a useful tool for ophthalmology residents and surgeons, and is currently utilized as a training device for surgeons and residents who are systematically learning cataract and vitreoretinal surgery. Training on the device prior to beginning live intraocular surgery is essential and has been proven to reduce intraoperative complication rates [3–5]. The validity of both vitreoretinal and cataract modules on Eyesi Surgical has been analyzed and simulator metrics confirmed [6–8]. Novice surgeons also show improvement in their intraoperative skills after training with Eyesi Surgical [9].

Basic abstract skill levels on the Eyesi Surgical platform, such as Navigation Training and Anti-Tremor Training, are not only valuable for ophthalmic surgery training. These skills can be transferred to a variety of microsurgical fields. The precision movements required for all types microsurgery can be practiced and repeated in the controlled, simulated environment.

The ability to practice these types of transferrable skills gives medical trainees insight into microsurgical fields of medicine and provides a preview of what a residency and future career in a microsurgical field might look like. To our knowledge, Eyesi Surgical has not been widely implemented in medical school curriculums. Feedback received after briefly introducing the simulator to other cohorts of students showed that simulator training was valuable hands-on experience. The students appreciated the opportunity to try out devices which allowed them to determine whether surgical interventions – mostly controlled on a microscope – are an option for them in their future career. The combination of feedback from the instructor as well as the simulator helped to provide students with real-time feedback about what subspecialties might be appropriate options for them to consider pursuing after medical school.

Based on this feedback, we chose to implement simulator training in a more structured way and adapted the training to be more generalized and applicable to student needs at their current learning stage.

The purpose of this study was to assess the inclusion of microsurgical simulation training in the medical school ophthalmology clerkship curriculum and determine the student response by means of subjective feedback from participating students.

## Methods

### Study design and investigation methods

This prospective, monocentric, exploratory study was approved by the ethics committee of the medical department of the Goethe University Frankfurt (resolution number E 205/19, transaction number 19–327) and was conducted at the Ophthalmology Department of the Goethe University Hospital Frankfurt. Participation in the study was voluntary. A total of 115 medical students in their 10th semester who had already attended ophthalmology lectures and were beginning their ophthalmology clerkship were included. Declarations of consent were obtained from all study participants before inclusion in the study.

Students were given the opportunity to attend two sessions with the Eyesi Surgical simulator (software version 3.4), on the first and last day of their ophthalmology clerkship. On the first day, students received an introductory lecture about the simulator technology and were given a short demonstration of how to work with it. This introduction was followed by a practical simulator training. Students began with a basic skills targeting task (level 1 of the Eyesi module ‘Navigation Training’) and progressed through a maximum of 5 additional modules over the course of the two sessions. As the levels advanced, they became more specific to ophthalmologic surgical technique. After completion of simulator levels, students were able to review a summary of their performance and results on the simulator, an example of which is shown in Supplementary material 1.

A survey tool consisting out of a ‘pre-simulation’ and ‘post-simulation’ – questionnaire (Supplementary Material 2) was developed and distributed to students. The survey was developed based on the principles of survey tool development which were discussed in a Medical Didactics course taken by the corresponding author at the Goethe University Frankfurt. Students completed a ‘pre-simulation’ paper-based questionnaire prior to using Eyesi Surgical at the first simulator session. They completed a second paper-based ‘post-simulation’ questionnaire at the end of session 1 or session 2, depending on if they attended one or both sessions. The supervisor of the course assured that each student responded only once and all questionnaires were pseudonymized. Students were asked to assign point values for each question. Questionnaire responses for self-rating of ability were scored on a scale of 0–10. A self-rated score of 0 corresponded with little to no ability and higher scores represented a higher self-perceived ability. Questions relating to the relevance or importance of simulator training were scored on a scale from 1–7. A score of 1 represented ‘strongly disagree’ while a score of 7 represented ‘strongly agree’.

### Acquisition of results/points from Eyesi Surgical

To evaluate the ability of the simulator to be used for general and abstract microsurgical skills, we chose to focus on the results from Navigation Training Level 1. The software module ‘Navigation Training’ consisted of abstract tasks that focused on microsurgical targeting skills in the human eye. Two instruments were available: a light probe and a straight needle. In level 1 of the module, 19 spherical objects were placed within the virtual eye. The spheres were approached with the tip of the needle. When the needle entered the sphere, the color of the sphere slowly changed from red to green. A bright green color signaled that the tip had been positioned for sufficient time inside the eye and the trainee could proceed to the next sphere. The trainee learned to move the tip of the instrument in a controlled and precise way. Rapid or uncontrolled approach with the needle causes the sphere to move away from the instrument and the trainee has to make a new approach attempt. The purpose of the module was to teach the trainee to carefully navigate the instrument to a specific location through a keyhole access within a closed environment under the microscope. For a video example of this task, see Supplementary Material 3.

A total score between 0 and 100 was calculated at the end of the module. Positive points could be acquired in the section Target Achievement. Negative points were awarded in the sections Efficiency, Instrument Handling, Microscope Handling, and Tissue Treatment. It was possible to acquire more than 100 negative points but this was not reflected in the total score which was truncated at 0 points. For each evaluation criterion, value ranges and points were defined to transform the measured value into a score. This was linearly interpolated according to the following formula:

$$relative\;value=\frac{value-start\;value}{end\;value-start\;value}$$

$$points=start\;points+relative\;value\ast\left(end\;points-start\;points\right)$$

Depending on the importance of a certain criterion, the range of achievable (positive or negative) points varied. Therefore, a single criterion could affect the total score only to a certain extent. For example, independently of how long a trainee operated out of focus, they got a maximum deduction of 20 points. In this case, it was still possible for them to achieve a total score of 80/100 points if every other task was performed perfectly.

Simulator criteria for Navigational Training Level 1 were sorted into 5 categories: Target Achievement, Efficiency, Instrument Handling, Microscope Handling, and Tissue Treatment. Each point earned for each criterion contributed equally to the total score (i.e. it was not weighted by category). Scoring type and range of points for each task, in addition to an example scored task, are listed in Table 1.

**Table 1:** Values and ranges of the scored criteria

Criteria	Scoring Type and Range	Range of Points	Example Scoring	
			**value**	**points**
**Efficiency**				
Time	80.. 280 s	0.. -20 pts	190 s	-11 pts
**Instrument Handling**				
Instrument slipped out of sphere	-2 pts/event	0.. -20 pts	3 events	-6 pts
Odometer	100.. 200 mm	0.. -20 pts	125 mm	-5 pts
Operating Outside Light Cone	-5 pts/event	0.. -20 pts	1 event	-5 pts
Operating Without Light Probe	-5 pts/event	0.. -20 pts	1 event	-5 pts
**Microscope Handling**				
BIOM Loupe Dipped into Visco		0.. -5 points	1 event	-1 pts
Operating Out of Focus	-5 pts/event	0.. -20 pts	1 event	-5 pts
**Target Achievement**				
Completed Objects	0.. 19 spheres	0.. 100 points	18 spheres	90 pts
**Tissue Treatment**				
Injured Retina Area	0.. 10 mm^2^	0.. -100 pts	0.5 mm^2^	-5 pts
Injured Lens Area	0.. 10 mm^2^	0.. -100 pts	0.5 mm^2^	-5 pts
Injured Macular Area	0.. 5 mm^2^	0.. -100 pts	0.5 mm^2^	-10 pts
Macular Spotted Hemorrhages	0.. 20 hemorrhages	0.. -100 pts	2	-10 pts
Phototoxicity	0.. 100 s	0.. -100 pts	3 s	-3 pts
Spotted Hemorrhages	0.. 50 hemorrhages	0.. -100 pts	2	-4 pts
**Total Score**		0.. 100 pts		15 pts

### Statistics

Data was recorded using the Eyesi Surgical platform and evaluated in Excel and in IBM SPSS Statistics v.28. A Spearman rank-order correlation coefficient was used to assess the relationship between simulator performance and self-rating of microsurgical skills. A Wilcoxon-matched-pairs test was used to evaluate the pre and post simulation training self-rating of microsurgical skills. A Kruskal–Wallis-Test was used to determine the relationship between residency specialty interest and simulator performance.

## Results

### Statistical evaluation

A total of 79 students could be evaluated based on questionnaire responses and simulator performance. 115 students began training with session 1 but 36 out of 115 did not complete both questionnaires and therefore were deemed lost to follow-up. Of the 79 students that completed both questionnaires and simulator training, 62 attended both sessions and 17 attended only one session. Some students were unable to attend both sessions due to scheduling conflicts with other curriculum requirements.

### Analysis of individual log values, Navigation Training Level 1

The average scores of individual evaluation criteria for Navigation Level 1 is displayed in Table 2. The mean Total Score was 25.7 (± 17.5) out of a possible 100 points. Target Achievement with Completed Objects was performed on average with relatively few mistakes. Tissue Treatment criteria were also completed with a low average point deduction. In contrast, activities in the Instrument Handling section resulted in a higher number of mistakes and point reductions. “Odometer” is an activity that quantifies instrument movement within the eye. Points are deducted from 0 for unnecessary movement. All students performed extensive instrument movements and therefore reached the saturation value of 20 negative points.

**Table 2:** Mean, minimum, and maximum scores for Navigation Level 1

Criteria	Mean Score	Min Score	Max Score
**Efficiency**			
Time	-19.6	-20	-10.5
**Instrument Handling**			
Instrument slipped out of sphere	-19.3	-20	0
Odometer	-20	-20	-20
Operating Outside Light Cone	0	0	0
Operating Without Light Probe	-0.19	-5	0
**Microscope Handling**			
BIOM Loupe Dipped into Visco	-0.11	-1	0
Operating Out of Focus	0 ± 0	0	0
**Target Achievement**			
Completed Objects	87.2	0	100
**Tissue Treatment**			
Injured Retina Area	-16.7	-100	0
Injured Lens Area	-0.4	-18.9	0
Injured Macular Area	-7.0	-100	0
Macular Spotted Hemorrhages	-6.1	-100	0
Phototoxicity	-0.5	-13.3	0
Spotted Hemorrhages	-1.7	-40	0
**Total Score**	25.7	0	49

### Evaluation of questionnaires

**Fig. 2:**
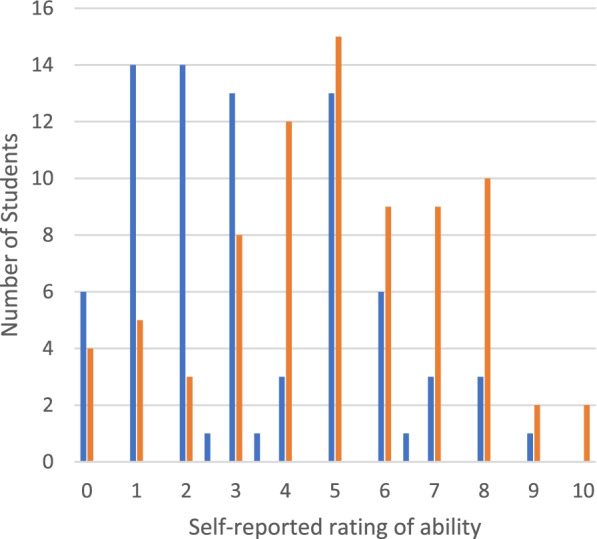
10^th^ semester medical students’(*n* = 79) self-rated microsurgical ability before and after training with Eyesi Surgical. Blue represents rating prior to training and orange represents rating after training. Students ranked themselves on a scale of 0–10, 0 meaning ‘little to no ability’ and 10 meaning ‘high ability.’

After evaluating the pre and post simulation questionnaires, it was found that the 73.4% of students self-rated their microsurgical skills higher after completing training with the simulator, when compared to their pre-simulation rating (*p* < 0.001) (Fig. 2). Mean self-rated score on the pre-simulation score was 3.3 (± 2.3) and mean self-rated score on the post-simulation questionnaire was 5.0 (± 2.4) (possible scores ranged from 0–10). A trend was found (*p* = 0.08) in the correlation between self-rated microsurgical ability and Navigation Level 1 total score on the Eyesi Surgical.

Students reported on the pre-simulation questionnaire which field they hoped to pursue during residency. Their answers were sorted into 3 categories, surgical specialty, non-surgical specialty, and undecided, based on if the specialty consisted of any surgical training during residency. Surgical specialties included general surgery, ophthalmology, vascular surgery, obstetrics and gynecology, urology, plastic surgery, oral and maxillofacial surgery, neurosurgery, orthopedics, pediatric surgery, trauma surgery, otolaryngology and urology. Non-surgical specialties included general medicine, internal medicine, anesthesiology, pediatrics, psychiatry, hematology, oncology, cardiology, neurology, radiology, forensic medicine, and pathology. 38% of the 10^th^ semester medical students were interested in a surgical specialty, 43% in a non-surgical specialty and 19% were still undecided.

There was no relationship found between students’ Eyesi Surgical performance scores and their pre simulator training specialty interests (*p* = 0.8).

**Fig. 3:**
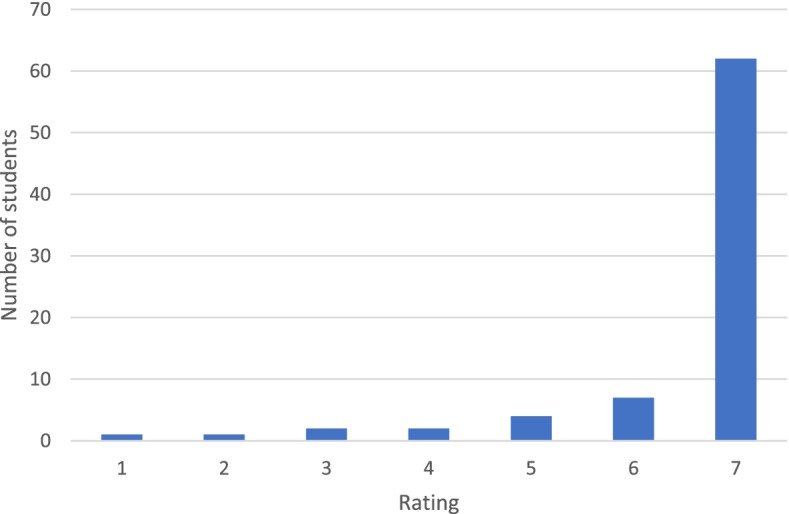
10^th^ semester medical students’ (*n* = 79) rating of the usefulness of Eyesi Surgical as part of ophthalmology clerkship as reported on the post-simulation questionnaire. Please note, a rating of 1 corresponded with ‘strongly disagree’ that the simulator is a useful component and a score of 7 corresponded with ‘strongly agree’ that the simulator is a useful component

92.4% of students reported that training with the Eyesi Surgical was a useful component of an ophthalmology clerkship during medical school, Fig. 3. 15.2% of students also commented on the questionnaire that they wished that there was more simulator training in the clerkship than was offered.

## Discussion

This study explored the subjective and objective results of ophthalmological microsurgical training for medical students. The microsurgical skill modules available on the Eyesi Surgical simulator, specifically those that are more abstract in nature, are useful for all medical students, not just those pursuing ophthalmology training. With the Eyesi Simulator, students are able to practice the precise movements that are required for microsurgery and receive immediate feedback. Our results showed that students achieved better scores on tissue treatment tasks than on instrument handling tasks. Students seemed to struggle in particular with the ‘odometer’ and ‘instrument slipped out of sphere’ criteria. These criteria relate directly to basic skills that must be acquired through extensive practice for successful surgery under a microscope. These results are not surprising as precision skills such as instrument handling take time to develop and all students in this study were complete novices.

Limitations to this study are the short training interval and a relatively high (*n* = 36) loss to follow-up rate. Loss to follow-up was attributed to curriculum conflicts which resulted in some students not attending their second planned simulator training day. These students were required to complete projects for a previous clerkship during their ophthalmology clerkship, which unfortunately resulted in a lower attendance rate on day two of simulator training.

Over recent years, there has been a decreased interest in pursuing general surgery residencies in many countries worldwide [10–13]. Exposing students to surgical skills by means of a simulator such as Eyesi may be helpful in providing a window into a future surgical career and boost interest in surgical subspecialties. The majority of students in this study reported that the Eyesi Surgical simulator was a useful component of their ophthalmology clerkship.

Students engagement, how to enhance it and how the result might have an effect onto the acceptance of a curriculum, the creation of innovative curricular changes and also the development of impactful extracurricular projects can be followed up in literature more frequently over the last 10 years.

In Germany, students in their 10.^th^ semester of medical school choose which elective specialty they would like to rotate through during their final year. Thus, giving students prior surgical experience with a simulator might help them to decide if they should consider a surgical specialty rotation. As the above results show, a number of students in this study were still undecided about which specialty they hoped to pursue after medical school. Considerations such as talent and enjoyment should not be minimized, and these are two factors that early introduction to basic skills with a simulator can help to clarify. Seo et al. and Yang et al. both reported increased medical student interest in surgical disciplines following a short surgical skills workshop [14, 15]

The importance of self-reported confidence in abilities and skills should not be overlooked when considering choice of specialty. Our results showed that a majority of students were more confident in their microsurgical abilities after completing training on the simulator. Similar results were reported by Antiel et al., who developed an intensive pre-clinical surgical experience for medical students. This one-week course, which included a simulation component, resulted in increased confidence in a variety of surgical skills as evidenced by self-reported abilities on pre and post experience surveys [16].

Kahu and Nelson [17] have summarized the understanding of mechanisms of student success and the meaning of student retention for higher education institutions. First, an educational interface adequate for the interaction between students and institutions is required. Second, psychological constructs including self-efficacy, emotions, belonging and well-being are essential for mediating the interaction between both students and institutions. Third, a concept to understand why some students with lower completion rates are retained and do go on to successfully complete their studies and others do not. This could help to explain the lower second session attendance rates in this study, which was mentioned as a limitation, and resulted from a conflict between several clerkships during the medical school curriculum. The design and implementation of curricula and co-curricular initiatives with different subspecialties considerations for each other are essential for a global success of education in medicine.

A meticulous debriefing with defined communication content (e.g. advocacy, inquiry, illustration, and confirmation) between debriefers and participants is mentioned by Berger-Estilita et al. [18] to be positively related to learning outcomes. Others, like Peters et al. [19], give suggestions for enhancing student engagement by maximizing dialogue between students and faculty. Zdravkovic et al. [20] stresses out that peer teaching, school governance and extracurricular activities lead to a high level of student engagement which also can affect innovative curricular changes and enable students to deliver highly impactful extracurricular projects.

The number of studies about students engagement potentially facing a shift in the teaching paradigms using simulation has increased over the last 15 years:

Okuda et al. [21] summarized in 2009 that simulation had become increasingly prevalent in medical school and resident education and that simulation is proven to be effective in the teaching of basic science and clinical knowledge, procedural skills, teamwork and communication as well as in assessment at the undergraduate and graduate medical education levels.

In educational scenarios let by a physical instructor, objective judgment and subjective feelings are united. The virtual trainer is completely free from subjective opinions, and provides clear objective feedback about student performance. As simulator software and technology continues to develop, this will become increasingly more optimized and fine-tuned to fit specific learning objectives. From our personal point of view, the stand-alone function has significant advantages, however the best training procedure includes an additional in-person instructor, who can provide hands-on assistance to the trainee at certain times during the training interval. This leads to the best results in satisfaction of the trainee as well as learning curve during the training process.

McGue [22] highlights the modern educational concept of simulation-based medical education. Essential experimental learning opportunities can be offered without risk to patients and simulation devices are differentiated into various categories: low-technology models, standardized “patients”, screen-based computer simulations, complex task trainers, high-fidelity patient simulators, and virtual reality systems.

Lu, Cuff and Mansour [23] summarize that because simulation is becoming an important tool in surgical education, faculty is being forced to modify their teaching of technical skill concepts. The authors recommend to concentrate teaching surgical skills ideally in a simulation center. Our personal experiences are that in such simulation centers which bring different medical subspecialties together in one place there are lots of advantages in regard to the infrastructure but we found also an imbalance between subspecialty representation: e.g. ophthalmology is considered to be a “small” subject with less need to be represented in these educational centres.

## Conclusions

Inclusion of the Eyesi Surgical Simulator in the medical school curriculum has the potential to bring additional ‘hands-on’ experience to medical students during their clinical rotations. Students have expressed interest in this type of experience, whether it be directly with patients or in a simulated environment. Practice on the simulator allows students to gain a better understanding of microsurgical techniques and additionally boosts self-confidence. Exposing students to surgical techniques early in their medical career can help to reduce the barriers that prevent students from pursuing a career in a surgical specialty. Practical experience and specifically surgical simulator experience should be integrated into the medical school curriculum to help better prepare students and help them find the right discipline that suits both their skills and interests.

## Supplementary Information

**Additional file 1: Supplementary Material 1.** Example of performance summary following completion of Navigation Training Level 1 on the Eyesi Surgical Simulato.

**Additional file 2:** **Supplementary Material 2. **‘pre-simulation’ and ‘post-simulation’ – questionnaire.

**Additional file 3:** **Supplementary Material 3. **video demonstration of ‘Navigation Training Level 1’ on Eyesi Surgical.

**Additional file 4:** **Supplementary Material 4.** Eyesi Surgical Navigation training data.

**Additional file 5:** **Supplementary Material 5. **Eyesi Surgical Questionnaire datasets.

## Data Availability

All data generated or analysed during this study are included in this published article and its supplementary information files.
