# Supplementary figures and images for: Implementation of microsurgery simulation in an ophthalmology clerkship in Germany: a prospective, exploratory study
Source: BMC Med Educ. 2022 Aug 3;22:599. doi: 10.1186/s12909-022-03634-x (PMC9351152; doi:10.1186/s12909-022-03634-x)

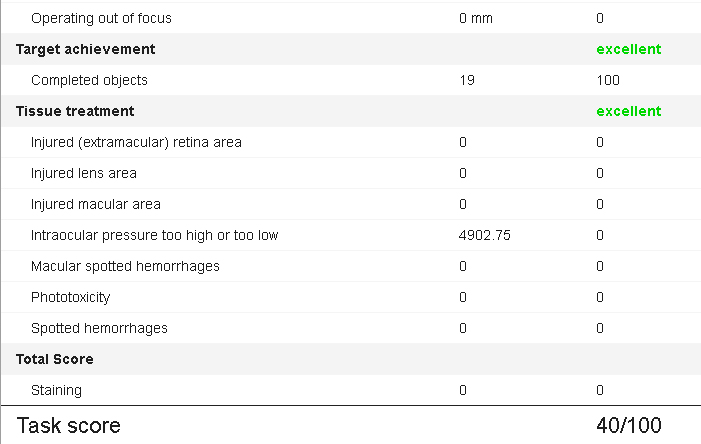

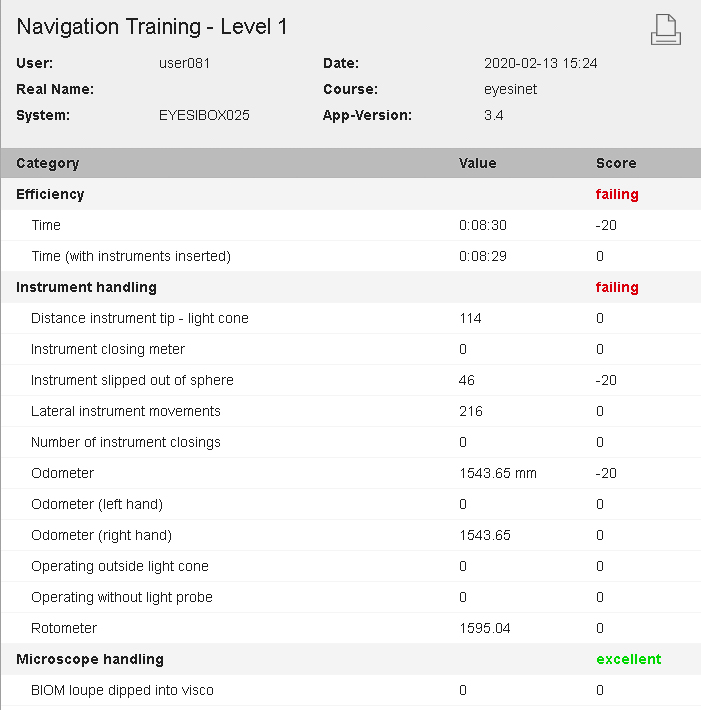

Supplement: Supplementary file 1 — Additional file 1: Supplementary Material 1. Example of performance summary following completion of Navigation Training Level 1 on the Eyesi Surgical Simulato. [file 12909_2022_3634_MOESM1_ESM.docx]
