# Supplementary material for: Implementation of microsurgery simulation in an ophthalmology clerkship in Germany: a prospective, exploratory study
Source: BMC Med Educ. 2022 Aug 3;22:599. doi: 10.1186/s12909-022-03634-x (PMC9351152; doi:10.1186/s12909-022-03634-x)
Supplement: Supplementary file 2 — Additional file 2: Supplementary Material 2. ‘pre-simulation’ and ‘post-simulation’ – questionnaire. [file 12909_2022_3634_MOESM2_ESM.docx]

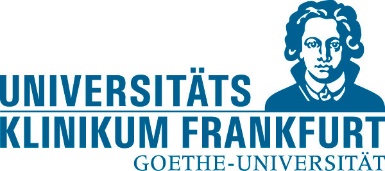


Department of Ophthalmology

**Director: Prof. Dr. T. Kohnen**

**Study Center Retina, House 8b**

Prof. Dr. F. Koch/ Dr. S. Deuchler

Tel. 069/6301-5689/-6459

Fax: 069/6301-5621

### Title of the Study

Evaluation of the efficiency of microsurgical simulation training in the context of student training

**Trainee Nr.:** ……………………………………..

**Before training with Eyesi Surgical:**

|  | Rating |
| --- | --- |
| On a scale from 0 (very bad) to 10 (very good): how would you rate your ophthalmological knowledge? | 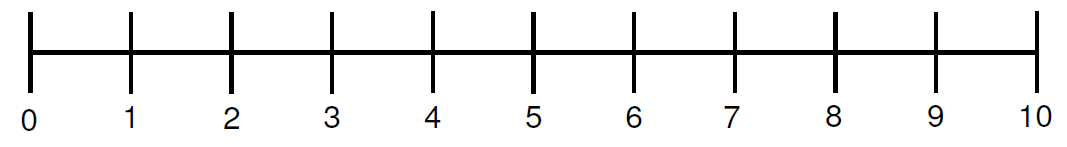 |

|  | Rating |
| --- | --- |
| On a scale from 0 (not interested) to 10 (very interested): how would you rate your interest in ophthalmology? | 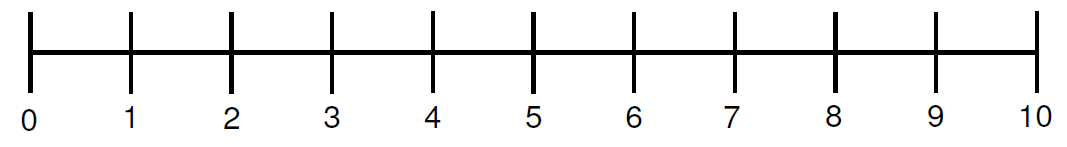 |

|  | Rating |
| --- | --- |
| How do you assess the relevance of ophthalmological disease for your later work (0 irrelevant, 10 very relevant)? | 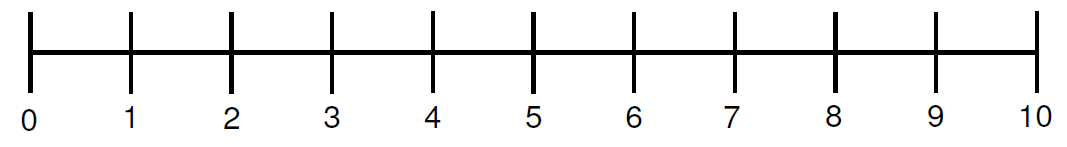 |

|  | Rating |
| --- | --- |
| On a scale from 0 (very bad) to 10 (very good): how would you rate your microsurgical skills? | 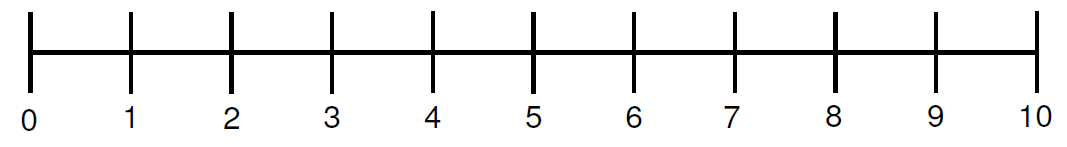 |

Which medical discipline would you like to specialize in?

**Thank you for your participation!**


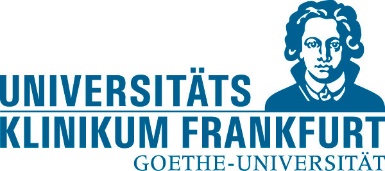


Department of Ophthalmology

**Director: Prof. Dr. T. Kohnen**

**Study Center Retina, House 8b**

Prof. Dr. F. Koch/ Dr. S. Deuchler

Tel. 069/6301-5689/-6459

Fax: 069/6301-5621

### Title of the Study

Evaluation of the efficiency of microsurgical simulation training in the context of student training

**Trainee Nr.:** ……………………………………..

**After training with Eyesi Surgical:**

|  | Rating |
| --- | --- |
| On a scale from 0 (very bad) to 10 (very good): how would you rate your microsurgical skills? | 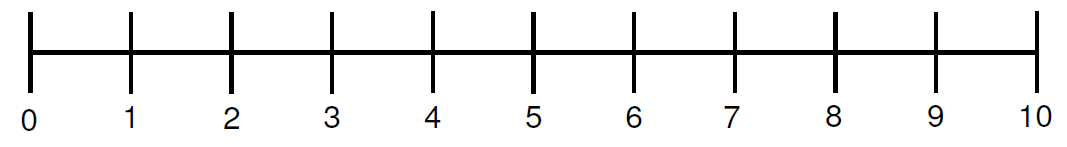 |

|  | Strongly Disagree  (1) | Disagree  (2) | Somewhat Disagree  (3) | Neither Agree nor Disagree  (4) | Somewhat Agree  (5) | Agree  (6) | Strongly Agree  (7) |
| --- | --- | --- | --- | --- | --- | --- | --- |
| Training with **Eyesi Surgical** has changed the self-assessment of my microsurgical skills. |  |  |  |  |  |  |  |
| The training with **Eyesi Surgical** has confirmed the self-assessment of my microsurgical skills. |  |  |  |  |  |  |  |
| Ophthalmology surgical training with the **Eyesi Surgical** is a useful part of the ophthalmology clerkship. |  |  |  |  |  |  |  |

**Thank you for your participation!**
